# Current Trends of Food Analysis, Safety, and Packaging

**DOI:** 10.1155/2021/9924667

**Published:** 2021-08-24

**Authors:** Bindu Modi, Hari Timilsina, Sobika Bhandari, Ashma Achhami, Sangita Pakka, Prakash Shrestha, Devilal Kandel, Dhan Bahadur GC, Sabina Khatri, Pradhumna Mahat Chhetri, Niranjan Parajuli

**Affiliations:** ^1^Biological Chemistry Lab, Central Department of Chemistry, Tribhuvan University, Kirtipur, Kathmandu 44618, Nepal; ^2^Department of Chemistry, Amrit Campus, Tribhuvan University, Leknath Marg, Kathmandu 44600, Nepal

## Abstract

Food is a basic necessity for life, growth, survival, and maintaining a proper body function. Rising food demand leads both producers and consumers to search for alternative food sources with high nutritional value. However, food products may never be completely safe. The oxidation reaction may alter both the physicochemical and immunological properties of food products. Maillard and caramelization nonenzymatic browning reactions can play a pivotal role in food acceptance through the ways they influence quality factors such as flavor, color, texture, nutritional value, protein functionality, and digestibility. There is a multitude of adulterated foods that portray adverse risks to the human condition. To maintain food safety, the packaging material is used to preserve the quality and freshness of food products. Food safety is jeopardized by plenty of pathogens by the consumption of adulterated food resulting in multiple foodborne illnesses. Though different analytical tools are used in the analysis of food products, yet, adulterated food has repercussions for the community and is a growing issue that adversely impairs human health and well-being. Thus, pathogenic agents' rapid and effective identification is vital for food safety and security to avoid foodborne illness. This review highlights the various analytical techniques used in the analysis of food products, food structure, and quality of food along with chemical reactions in food processing. Moreover, we have also discussed the effect on health due to the consumption of adulterated food and focused on the importance of food safety, including the biodegradable packaging material.

## 1. Introduction

The major classes of nutrients are primarily obtained from food, including essential amino acids, organic acids, and peptides which cannot be generated in the human body [1]. Food also brings various health problems when consumed in an inappropriate quantity or without considering a balanced diet. Improperly consumed food cannot be beneficial and cause a severe problem in health. One major aspect of food quality is the contamination of food by other harmful constituents. For food consumers, the examination of food contaminants and their quantitative analysis is of paramount importance. Thus, continuous assessment of food quality and its safety is essential to deal with public health [2]. Food hazards are another focused area in the changing contexts. Thermal processing quickly removes biological and physical hazards rarely causing significant foodborne diseases [3]. Several cases of chemical hazards have been identified, such as melamine contamination in infant formula powder, heavy metal residues in fish products, and egg contamination with insecticide fipronil, which have a direct effect on consumer health [4]. Chemical analysis of food constituents allows one to assess food quality in such respect [5]. The chemical hazards or contaminants present in food are detected by spectroscopic techniques such as mass spectrometry, ultraviolet detection, fluorescence techniques either alone or in conjunction with other separation techniques, thermal processing, electrophoresis, and specific quantification immunoassays [6, 7]. The method of analysis also uses chromatographic techniques such as gas chromatography and high-performance liquid chromatography [8]. However, several factors may directly influence foods' chemical properties, resulting in variations in their bioactive and nutritional qualities. Thus, the implementation of efficient, versatile, and reliable analytical techniques to assess the authenticity and traceability of foods is of keen interest [9].

The issue of food security at present has set a daunting task for the food industry. In addition to an ever-expanding world population, the requirement for food is increasing, generating a greater and more complex food chain. However, the food may be contaminated by parasites, toxic agents, bacteria, and pathogens, as well as pollutants that bring more than 200 diseases, including severe infectious diseases and even cancers [10]. Thus, food safety is a major public health concern for the food processing and packaging industries, distributors, retailers, and consumers, as every year, about 48 million people get sick, about 1.28 million people get admitted to hospitals, and about 3000 people die due to consumption of unsafe and adulterated food products [11]. The principal aim of food assessment is associated with the maintenance of food safety and security. Food laboratories swap their conventional technologies for innovative and modern analytical methods to pursue new visions [12, 13]. One of the prevailing analytical challenges in food product safety is accurately portraying precise information as efficiently as possible concerning official guidelines without impeding the attributes of procedures such as precision, recovery, and sensitivity [14]. There are still many problems that need to be addressed in authentication and traceability of food, with a considerable range of analytical innovations and implementations seen in food analysis [15].

The safety of food products is intrinsically linked to the method of food packaging. Moisture, heat, and microorganisms often pollute food, and its quality is diminished. Microorganisms contribute to the threat of diseases transmitted to food among people and thereby pose a public health concern. Thus, the packaging material is used to avoid food contamination and reduce food waste and preserve food quality [16]. Plastic packaging materials represent a vulnerability to the ecological system since they are not degradable in the environment. Therefore, today's society needs both environment-friendly and degradable packaging materials. Scientists and researchers worldwide have been intrigued by the adoption of biodegradable plastics for food packaging [17].

## 2. Current Status of Food

### 2.1. Food Structure and Quality

Foods are composed of nutritionally essential components that majorly include carbohydrates, proteins, fats, vitamins, water, and minerals as well as fiber. Foods are regarded as “functional” based on their composition as it is responsible for the quality of the food determining the health benefits [18]. Starch, a significant source of energy for humans, produced in storage units of plants, consists of almost completely two main polysaccharides (amylose and amylopectin) [19]. The relation between starch structure and digestion is highly complex, and the starch in digestion extent and speed can also get affected by the amylose content, amylopectin structure, degree of crystallization, and particle size [20]. The majority of starchy foods are however poor in dietary fiber (DF). DF is the edible portion of the plants that are nondigestible carbohydrates and therefore proceed to the bowel. Polysaccharides, oligosaccharides, lignin, and related plant substances constitute dietary fiber [21], and intakes of the meal with an adequate amount of DF are proved to be crucial in reducing the risk of various diseases like heart diseases, high blood pressure, diabetes, obesity, cancer, and other gastrointestinal disorders [22].

Food structure is decided by the different ingredients present and the processing of producing food. In terms of ingredients, various bioactive materials, color, and flavor enhancers as well as functional elements are now being introduced into the dietary system [23]. A best natural source of antioxidant, antimicrobial, anti-inflammatory, antimutagenic, and antiviral activity, as well as other health-friendly properties is a structurally diverse range of bioactive compounds like carotenoids, polyphenols, flavonoids, vitamins, organic acids, phytosterols, nucleosides, and fatty acids extracted from various plants and microorganisms (algae, bacteria, fungi, and myxomycetes) [24, 25]. Thus, in utilization of these extracted bioactive molecules into the food system to make the food qualitative and functional, interests and efforts are being given worldwide. The substantial amount of bioactive compounds in beetroot pulp can be used to create a variety of novel value-added functional foods to fight diseases such as diabetes, cardiovascular disease, cancer, and other chronic diseases while also being widely utilized in the food industry as a nontoxic food colorant or additives [26]. No doubt, bioactive compounds in the food system play a crucial role in determining the quality of the diet. However, most of the active compounds are water-insoluble that creates difficulties in the absorbance of the active constituents so the bioavailability to the desire sites has always been a challenge as their bioefficacy depends upon the chemical structure and the matrix of food [27].

Processing can positively or negatively modify both nutrient density and food structure (the matrix effect) that determine food health potential [28]. Food structure gets changed due to food processing, determining various appearances, tastes, texture, and food functionality. For instance, the food drying method is aimed at eliminating water to prolong food shelf life deteriorates food quality. However, this change in product quality attributes like shrinkage, deformation, and color was observed less in the rugged surface, indicating better textural characteristics and more quality in it than in the plane surface [29]. Basically in cooking, the starch, amylose, and resistant starch content lowered, and the sugars increased. Cooking methods also vary the sugar contents as on boiling, microwaving, and frying; starch content decreased substantially on average by 40%, 64%, and 2% and amylose by 14%, 17%, and 34%, respectively [30]. Freezing is the most used food storage method during which cellular food moisture is crystallized and made into ice crystals, which are most likely to negatively impact the microstructure of food and contribute to the loss in quality of food on thawing [31]. The consistency of frozen vegetables can also be influenced positively by chemical pretreatment. The effects of the addition of soybean protein isolate, whole milk powder, and sodium caseinate in mashed potatoes and then frozen for at least one month at ₋24°C confirmed the positive results on rheological properties among which soybean protein isolate showed greater ability to minimize the freeze effect [32]. Hydrolyzed egg yolk protein has been reported as a novel additive to prevent irreversible gelation induced by the freeze/thaw process better than NaCl or sucrose [33]. In addition to that, microorganism fermentation enhances the nutrition quality by increasing the content of bioactive compounds along with short-chain fatty acids through the metabolism of food components like fiber and carbohydrates [34]. One of the significant factors in deciding food quality, which is regarded as the sensory property of food, is food texture that arises from mechanical and structural elements and is capable of being tested only by humans [35]. Food structure is related to mechanical properties. Consequently, rheological changes are related to structural changes. Rheological activity not only has a significant effect on the development, storage, and texture of hydrogels of proteins/polysaccharides but also naturally represents the molecular structure and chain conformation of macromolecules [36].

Food structure designing with the inclusion of sufficient amounts of desirable nutrients retaining or improving the textural/sensory effect and quality of food is of great interest to all [37]. The involvement of dietary fibers and pullulans has demonstrated the interference effect in starch hydrolysis by reducing gelatinization by sequestering some water, covering the starch granule, and increasing the viscosity of the gel [38]. Thus, the addition of DF and the water-soluble polysaccharide polymer and pullulan increases the quality of food by reducing the risk of obesity and diabetes by limiting starch absorption. In the water phase or the oil phase, several efforts have been taken to develop gel structures for enhancing the bioactive compounds' delivery efficiency and several bioactive food ingredients be stable and bioavailable [39]. Food structure is decided by the composition of the food that plays a vital role in determining its quality and health benefits.

### 2.2. Effect of Pesticides on Food and Detection Methods

In this modern era, pesticides are often used, particularly to preserve food, and in the agrarian and floricultural practices, to combat undesirable pests and improve productivity which has undoubtedly reduced food value. The residue of such chemicals on soil, terrestrial, and aquatic ecosystems affects not only human health but also animals' health [40]. For instance, organochlorine is the most toxic yet widely used pesticide in agriculture, and its exposure has caused a dramatic decline in honeybee populations as well as direct pollution of honey [41]. To detect organochlorine pesticides like DDT chemically known as 1,1′-(2,2,2-trichloroethane-1,1-diyl) bis (4-chlorobenzene), an immune nanobiosensor based on AuNPs in dipstick format using competitive immunoassay was developed [42]. Furthermore, in Israel, all honey samples were regularly identified with at least two pesticides, amitraz metabolites and coumaphos, and at most nine more, while lipophilic pesticides were primarily contained in beeswax, and the results suggest that children's well-being can be jeopardized if honey and beeswax are consumed regularly [43]. 24 new pesticide residues were selectively determined by a miniaturized extraction-partition procedure requiring small amounts of nonchlorinated solvents in fruits like apple puree, concentrated lemon juice, and tomato puree [44]. New detection methods like aptasensor [45], enzyme-based biosensor [46], origami multiple paper-based electrochemical biosensors [47], and graphene quantum dots-based photoluminescent sensor [48] are used for the detection of pesticides in food. Along with the advancement of spectrometric methods, liquid and/or gas chromatography-tandem mass spectrometry (LC-MS/MS and/or GC–MS/MS) and biosensors are used for monitoring pesticides, and various enzyme inhibition-based detectors have been used for detecting pesticides in food [49, 50]. To ensure food safety, pollutants must be continuously regulated and monitored using appropriate advanced procedures.

### 2.3. Adulteration in Foods and Health Risks

Food adulteration is the act of intentionally degrading the quality of food offered for sale either by adding or substituting inferior materials or by the removal of some valuable ingredients. Just for limited economic advantages, foods are being adulterated without caring for an individual's health [51].

The current issues on the food show that no food is deprived of food adulteration. Melamine, a nitrogen-rich compound and 67% nitrogen per mass unit, is adulterated in milk and wheat gluten to increase protein content and avoid detection, as milk was tested based on nitrogen content. In India, sample nonconformity was 31% out of 81% of unpacked milk samples in the rural area. In the urban area, 8% of detergent, 45% of skimmed milk powder, and 27% of glucose were found in the unpacked (67%) nonconforming sample [52]. There are many examples of the adverse effect of food adulteration on human health. Some of them are mentioned as (i) paralysis, cancer due to the addition of minerals oils in edible oils and fats; (ii) abortion, brain damage of baby, liver damage, and allergies; (iii) stomach disorders, giddiness and joint pain by the substitution of coffee powder with chicory powder; (iv) vomiting and diarrhea because of zinc substances and so on [53].

Various techniques (physical, chemical/biochemical, and molecular) are applied for the detection of adulteration based on the type of adulteration to be detected. Physical techniques include macroscopic visual structural analysis and analyzing the physical characteristics of food [51]. Chemical/biochemical techniques include spectroscopic, chromatographic, immunologic, and electrophoretic-based techniques [54]. Some methods like vibrational spectroscopies, near-infrared, Raman, NMR spectroscopy, midinfrared, and mass spectrometry techniques are developed not only to concern the continuous engagement with adulterated food but also on serious issues like food security, bioterrorism, and climate change [55]. Initially, thin-layer chromatography [56], adsorptive voltammetry [57], and spectrophotometry methods [58] are used to detect food dyes and are now replaced by capillary electrophoresis [59], reversed-phase liquid chromatography (RPLC), and ion pair RPLC since they are time-consuming and are not suitable for color mixtures [60]. Food preservatives like formaldehyde used in aquatic products are detected from surface-enhanced Raman spectroscopy Au/SiO_2_ used as enhancer substrate [61]. Nitrate and nitrite in cured meat are detected by sequential injection analysis and benzoate and sorbate in orange beverage and tomato by concentrated gas chromatography [62].

UV-visible, midinfrared, and fluorescence spectra are used for testing the purity and adulteration of pomegranate oils [63]. The combination of visible-near infrared spectroscopy (Vis-NIRS) with chemometric tools like hierarchical cluster analysis (HCA), principal components analysis (PCA), and linear discriminant analysis (LDA), is used for the detection of high fructose corn syrup in honey [20], and detection of adulteration of fresh olive oils with old olive oils has been done by the combination of midinfrared, UV-visible, and fluorescence spectroscopy [63], in grape syrup by dielectric spectroscopy sensors (parallel plate capacitor (PPC) and cylindrical stub resonator (CSR) [64]. FTIR and ATR spectroscopy were explored to discriminate between beef meat and chicken meat [65]. Raman spectroscopy is used to detect titanium dioxide (TiO_2_) which is used as a color additive in food. The combination of FTIR technique and chemometric methods provides effective cooperation in tracing and detecting adulteration [66]. Enzyme-linked immunosorbent assay (ELISA) is an immunological approach used for the detection of food adulteration. Similarly, adulteration in milk can be detected by urea-PAGE in terms of the origin of milk's species [51].

Nanotechnology has great potential in detecting food contamination (biological and chemical contaminants) through nanosensors and nanobiosensors. For example, a sensitive electrochemical AChE nanobiosensor developed on PANI and MWCNT core shell-modified GCE is used to detect carbamate pesticides in fruits and vegetables [67]. Carbofuran (a pesticide used to control insects and nematodes on crops) is detected and qualified by using AuNPs, Prussian blue-MWCNT-CTS (PB-MWCNT-CTS) nanocomposite film, and staphylococcal protein A (SPA) layer-by-layer assemble technology (electrochemical immunonanosensor) [68]. Melamine used in milk as adulterated is detected by the combination of AuNPs, cadmium telluride (CdTe) quantum dots (QDs), and SWCNTs with various sensors rapidly [69, 70].

Though we all know “health is wealth,” people with an economically motivated mentality do not even leave food by making food adulterated. Adulterated and contaminated food has been a part of our daily life because adulteration is everywhere even though various acts on food adulteration have been implemented. That is why we should be aware of the adulteration process and mostly use organic ones for our healthy life.

### 2.4. Mycotoxins and Food Contamination

Mycotoxins are low molecular weight, secondary toxic metabolites synthesized by fungi such as *Aspergillus*, *Fusarium*, and *Penicillium* that cause food and feed contamination globally inviting several health hazards [71]. Among several renowned mycotoxins, aflatoxin, ochratoxins, fumonisins, and their derivatives are the most common toxicologically familiar mycotoxins [72]. According to a WHO survey, aflatoxin contamination affects about 25% of the world's food crops causing major economic loads [73]. The intake of mycotoxin-infected foodstuffs causes serious health problems to humans and animals that can be carcinogenic, mutagenic, teratogenic, nephrotoxic, hepatotoxic, embryotoxic, and immunosuppressive [74, 75]. Mycotoxin contamination, mostly by aflatoxin, fumonisins, ochratoxins, and deoxynivalenol is observed in major agriculture crops like cereals, groundnut, milk, coffee, and beer and also appears in entire animal fodder and human foodstuff [76]. These food cause chronic intoxication relative to acute symptoms. High-dose mycotoxin exposure results in acute toxicity like abdominal pain and diarrhea, while low-level mycotoxin exposure for prolonged period results in severe damage in the liver, kidney, and immune system organs causing cancers in these organs [77].

### 2.5. Nutrition and Alternative Diets

Food quality refers to food, acceptable by consumers with the main characteristics of having safety, nutrition, freshness, availability, convenience, and integrity [78]. Biogenic amines (a nitrogenous and organic compound) like tyramine, histamine, and spermidine are found in a wide range of food (cheese, wine, meat, vegetables, fish, etc.), with different concentrations. Biogene amine histamine is one of the toxins targeted by the Food and Drug Administration (FDA) and European Food Safety Authority (EFSA) and also plays an important role as indicator of food quality [79]. The quality of meat and flesh is determined by the presence of proteins, essential amino acids, essential fatty acids, vitamins (A, E, and B), and minerals [80]. Because of the high content of glycine and nonessential amino acids, collagen-rich muscles have a lower nutritional value [81]. LED green light technique could help extend shelf life, maintaining visual quality, increasing DPPH radical scavenging activity, and preventing the decrease of bioactive compounds in broccoli florets [82]. A novel hydrothermodynamic (HTD) technology, based on the high turbulence and cavitation in viscous liquids, has the potential for manufacturing originative natural whole food with high nutritional and nutraceutical values. It has the potential to minimize the effect of thermal degradation of bioactive phenolics and increase the shelf-life of pasteurized blueberry food [83].

To meet the balanced diet, an alternative diet is of great importance and can be included in the diet by various methods. Vitamin D can be synthesized from cholesterol under the skin in the presence of UV light but can also be consumed from the diet through fish, eggs, fortified milk, and mushrooms. The active form of vitamin D and calcitriol (1,25 D dihydroxy vitamin D), developed after hydroxylations in the kidneys and liver, has been shown to regulate the immune system [84]. Vitamin D reduces the risk of viral infections. Grant et al. discussed data supporting the position, including those from influenza, coronavirus (CoV), and pneumonia, for greater concentrations of 25-hydroxyvitamin D (25 (OH)D) in reducing infection and death risk from acute respiratory tract infection (ARTIs). Research indicates that increased intakes of vitamin D can decrease infection risk and COVID-19 [85]. The water-soluble nutrient vitamin C serves as an antioxidant that scavenges reactive oxygen species (ROS), thus shielding biomolecules from oxidative damage and dysfunction, such as proteins, lipids, and nucleotides. In leukocytes, vitamin C accumulates at levels 50-100 times higher than in plasma, which can demonstrate the vitamin's functional roles in these immune system cells [86, 87]. Foods like Kakadu plums, cherries, chili peppers, guavas, kiwi, broccoli, and citrus fruits are rich sources of vitamin C, which exhibits a plausible mechanism of anti-inflammatory and immunomodulatory functions that are important to severe respiratory infections [88].

Vitamin B helps to better stimulate both innate and adaptive immune responses, decreases proinflammatory cytokines, and enhances respiratory function. Vitamin B plays a crucial role in cells' functioning, energy metabolism, and proper immune function [89]. A combination of vitamin D/magnesium/vitamin B_12_ in older patients with COVID-19 was associated with a substantial reduction in the proportion of clinically affected patients needing oxygen support, intensive care support, or both [90].

Vitamin E can be obtained through dietary sources such as vegetable oils, nuts, seeds, and various leafy vegetables and fortified cereals. In animal and human models, vitamin E has been shown to strengthen immune responses and provide defense against many infectious diseases. By scavenging oxygen species, vitamin E can exert its immune-enhancing effects to reduce oxidative stress [91, 92]. Healthy dietary choices with high biological value protein intake, such as fish, eggs, lean meat (poultry), and whey protein (or other nonfat dairy protein), can decrease inflammation and postprandial lipogenesis when consumed together with meals [84]. To survive, proliferate, and work, immune cells depend primarily on amino acids' availability like glutamine and ultimately protect our body against pathogens. Metabolism and immune functions are modulated by certain amino acids such as glutamine and l-arginine [93, 94].

Among polyunsaturated fatty acids (PUFAs), arachidonic acid (AA), eicosapentaenoic acid (EPA), and docosahexaenoic acid (DHA) are essential long-chain PUFAs. To preserve human health, dietary intake of AA, EPA, and DHA is important, and livestock and marine foods are the primary sources of nutrients for AA and EPA/DHA, respectively [95]. AA is nontoxic and can be administered orally and intravenously, and its administration increases the formation of lipoxin A4 (LXA4), an anti-inflammatory enzyme that also has AA-like antiviral and antibacterial activities [96]. This is supported by reports that exogenous linoleic acid (LA) or AA supplementation in human coronavirus- (HCoV-229E-) infected cells substantially suppressed the replication of the HCoV-229E virus and preserved the inhibitory effect of LA and AA on virus replication for the highly pathogenic coronavirus respiratory syndrome in the Middle East (MERS-CoV) [97]. SARS-CoV-2, SARS, and MERS are enveloped and can quickly be inactivated by AA and other unsaturated fatty acids. Oral or intravenous administration of AA and other unsaturated fatty acids is recommended to enhance tolerance and recovery from such infections [96].

Zinc is a trace element with immunoregulatory and antiviral properties, found in various fruits and vegetables. For cell growth and immune cell maturation, zinc is important, particularly in T-lymphocytes' development and activation [98]. Malnutrition is the principal cause of zinc deficiency. Zinc deficiency leads to cell-mediated immune disorders. Zinc deficiency encourages the development of proinflammatory cytokines and is associated with fibrosis-predisposing inflammatory alterations throughout the lungs. Iron, an enzyme component critical for immune cells' function, is important for cell differentiation and development [99]. Iron-deficient children are more likely to develop persistent acute respiratory tract infections (ARTI) [100]. Anemia is prevalent in patients with serious infection with SARS-CoV-2 and that anemia is associated with extended hospital stays, poor health outcomes, and poor survival [101].

The diet we take regularly might not be available every time, so one should have a sound knowledge of alternative diets. A healthy diet, compliance with safety conditions, and finding appropriate and safe methods to increase the body's immunity, is an excellent alternative to a major transition through difficult times, such as pandemics [102]. The bioactive peptides extracted from food have gained increased interest as agents in chronic disease control and reduce the risk of side effects resulting from synthetic drugs. Bioactivity associated with cereal proteins includes antioxidant, anti-inflammatory, cholesterol-lowering, satiety, antidiabetic, and others recently studied [103].

Growing food demand would cause producers and consumers alike to look for alternative food sources of high nutritional value; snails, in particular, may be considered a good candidate to avoid viscera containing high concentrations of nonessential trace elements, such as Al, Cr, Cd, and Pb. An interesting and alternative source of critical trace elements, such as Fe, Zn, Cu, Mn, and Se, may be considered the home-processed food analyzed in this research. In this analysis, concentrations of eight important (Fe, Zn, Cu, Mn, Se, Ni, Mo, and Co) and six nonessential (Pb, Cd, Hg, Al, As, and Cr) trace elements were determined in home-processed food derived from snails and three common species of game animals (woodcock, pheasant, and hare), seasoned with anchovies, mushrooms, and various vegetables using inductively coupled plasma mass spectrum (ICP-MS) [104].

The human immune system is very important for a normal existence and can prevent the mass spread of major health problems, such as introducing novel viruses, such as COVID-19, which has rapidly become a global pandemic. Stress is detrimental to human health since it produces free radicals in the human body. According to numerous recent reports, volatile oils from different aromatic plants have high antioxidants and antimicrobial substances. To kill these free radicals, an external supply of antioxidants is needed [103]. Thus, to meet the ever-increasing food demand, above-mentioned alternative diets and nutrition can be included in the food table.

## 3. Analytical Approaches

### 3.1. Chemical Reaction in Foods

The Maillard reaction (MR) is defined as an array of nonenzymatic, consecutive, and parallel chemical reactions that supervise both food quality and safety. Since Louis-Camille Maillard observed in 1912 that a mixture of amino acids and sugars resulted in a brown solution upon heating, overwhelming evidence established that the condensation reaction between reducing sugars and amino groups of free amino acids or proteins is the main source of N-glycoside derivatives in foods and *in vivo* [105]. Maillard reaction is a part of the nonenzymatic browning reactions. Due to the formation of caramelization, the reaction of polymers called melanoidins produces a characteristic brown color. For the food industry, this is a critical reaction, as it describes a large part of the sensory properties, fragrance, and taste of the products cooked. In terms of anti/prooxidant ability, immunogenicity, allergenicity, and carcinogenicity, Maillard reaction products (MRP) can have beneficial or adverse effects on health [106]. Maillard reaction products (MRPs) result from a chemical reaction between amino acids and sugar reduction when foods are processed at high temperatures. This reaction increases flavor and color, and positive and adverse health effects have been correlated with MRPs [107]. Maillard reaction takes place through multiple reactions, which can follow several different routes. However, the reaction pathway can divide the reaction sequence into three primary stages. The scheme of formation of melanoidins from aldose sugar is shown in the supplementary section (Figure S1) [108].

**Figure 1:**
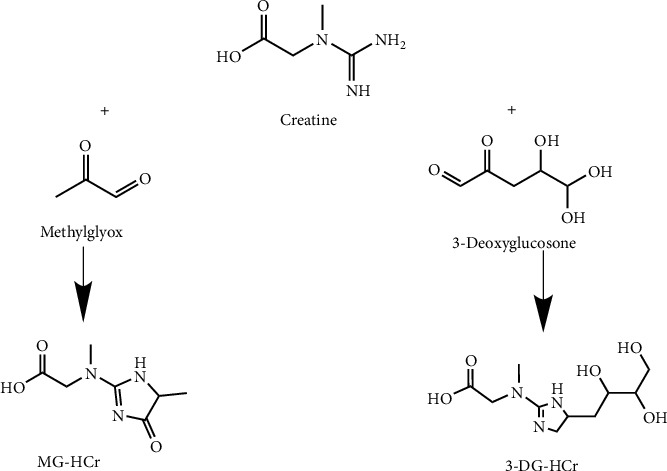
Formation of MG-HCr and 3-DG-HCr from the reaction of creatine with methylglyoxal and 3-deoxyglucosone, respectively [109].

Some of the key molecules are depicted on the top, the Amadori compounds, in the middle Strecker's aldehyde, dicarbonyls, and at the bottom, acrylamide, styrene, and melanoidins. The chemical reactions that changed due to the Maillard reaction have been studied in the following food processing. Methylglyoxal (MGO) and 3-deoxyglucosone (3-DG) are 1,2-dicarbonyl compounds formed from carbohydrates during caramelization and the Maillard reaction, Figure 1. Under physiological conditions, MGO is also formed as a byproduct of glycolysis, and 3-DG is formed from 3-phosphorylated fructose and fructosamine [109].

Caramelization is a nonenzymatic browning reaction of sugars providing a caramel-like flavor during the high-temperature treatment of foods. Sugar degradation during the Maillard reaction, characterized by nitrogen-containing low and high molecular weight compounds, is catalyzed by amino acids. Both reactions proceed together at elevated temperatures so that one affects the other. The Maillard reaction may take place under milder conditions, but sugars are caramelized at temperatures above 120°C [110].

**Figure 2:**
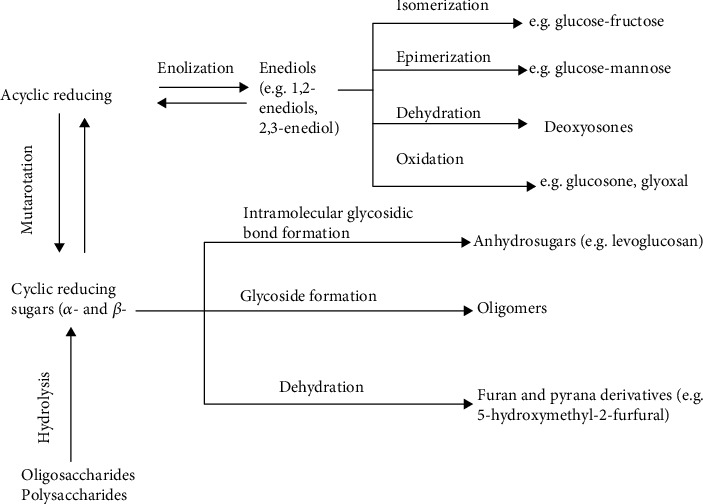
Main reactions of sugar degradation [110].

The main reactions of sugar degradation are schematized in Figure 2. The acyclic sugar forms are very reactive, and by increasing temperature, more acyclic forms are discovered. Ring-opening followed by enolization initiates isomerization, epimerization, dehydration, and oxidation reactions of the cyclic reducing sugar.

Likewise, the physical and chemical properties of thermally and oxidatively degraded sunflower oil and palm fat are subject to a variety of changes. A significant method used worldwide for the preparation of foods is deep-fat frying. Numerous polar compounds are formed due to oxidation, hydrolysis, decomposition, and oligomerization. These compounds modify the physical, nutritional, and sensory properties of oil or fat [111].

Similarly, cholesterol oxidation products, cholesterol dimers, and cholestadienes are formed after thermal processing of cholesterol standards and butter. In particular, the presence of cholesta-3, 5-diene is examined in unheated samples of free cholesterol and cholesteryl palmitate (105.3-116.4 mg g^−1^) and thermally processed butter (0.009 mg g^−1^). Moreover, standard samples (34.7-98.8% losses) relative to butter samples (25.5-73.5% losses) observe the processes of extensive cholesterol degradation. Research indicates that cholesterol-containing materials' thermal processing should be carried out at the lowest possible temperatures, e.g., 150°C, which prevents cholesterol from dimerization, oxidation, and degradation [112].

Similarly, there is an effect of temperature on acrylamide formation in cocoa beans during drying treatment. When foods containing free asparagine and reducing sugar are cooked at a temperature above 120°C in low humidity conditions, acrylamide is produced [113]. During the production of block panela (noncentrifugal cane sugar), Mesias et al. evaluated the formation of acrylamide and other heat-induced compound hydroxymethylfurfural (HMF) and furfural at different levels [114].

### 3.2. Current Status of Analytical Testing of Foodstuffs

A huge number of analytical techniques have been used for the food analysis; few of which include (a) spectroscopic as mass spectrometry, nuclear magnetic resonance, infrared, etc.; (b) biological as a polymerase chain reaction, biosensors, etc.; (c) separation as high liquid performance chromatography, capillary electrophoresis and so on [13]. Among those varieties of analytical techniques, the chromatography technique can be considered as the best method for the simultaneous determination of several classes of contaminants and residues. In the last few years, though gas chromatography has been used to determine nonpolar compounds, LC has been widely used for monitoring the more polar compounds. HPLC is becoming a good choice due to its considerable reduction in the analysis time [115]. But several problems such as strong matrix effects and low retention time make it not amenable to multiresidue methods, and to reduce analysis time, the authors have proposed eliminating chromatographic steps and using flow injection analysis [116]. The use of hydrophilic interaction chromatography is another alternative for the determination of very polar compounds [117]. Nowadays, the miniaturization of the chromatographic systems (micro- or nano-LC) has been widely used in proteomics, offering suitable properties as robustness and reliability, as well as for the determination of allergens, amines, pesticides, and toxins in food since it possesses powerful possibility to reduce sample volume and analysis time, increasing sensitivity, separation efficiency, and peak capacity [109, 110].

To confirm the screening result, chromatographic separation is not enough, and confirmatory methods require a mass spectrometric detector. The commonly used methods that enable wide linear ranges and LODs down to the microgram kg^−1^ level or even less are liquid chromatography-tandem mass spectrometry (LC-MS/MS) and gas chromatography-tandem mass spectrometry (GC-MS/MS) [118]. However, the QqQ detector coupled with chromatographic systems provides a great result for hundreds of analytes per run whereas chromatographic methods coupled to HRMS may also be used due to their high-throughput and excellent selectivity for food contaminant screening [119]. Instead of the classical reversed-phase (RP) systems for the separation of polar analytes, alternative LC separation techniques such as hydrophilic interaction liquid chromatography (HILC) and supercritical fluid chromatography (SFC) have been proposed although C18 or octyl (C8) columns can efficiently separate nonpolar compounds; polar analytes separation can be a rather challenging task. HILC follows the opposite mechanism of RP, that is, the polar stationary phase retains polar analytes that are eluted by a mobile phase consisting of a mixture of acetonitrile (usually) and water [120]. Hence, the strongest weapon to detect contaminants in foodstuffs is the chromatographic separation coupled to various MS detectors. For the analysis of phthalates, BPA, and NIAS in food, and food packaging materials, the mainly used technique is GC-MS [121]. On the other hand, for the more polar substances including PFCs, PAAS, and photoinitiators, LC-MS/MS has been the selected technique [122]. The application of HRMS in LC for target, posttarget, and nontarget analysis has been observed but not in the case of GC. Recently, for the analysis of some FCM contaminants such as PFCs, PAAs, additives, and phthalates in FCMs, the LC-HRMS method using TOF or Orbitrap mass analyzers has been introduced. Applying the HRMS technique, the analytical challenges in this field should be the development of FCM contaminants methodologies in food, food simulants, and food contact materials [123]. The presence of mycotoxins is determined by chromatography like high-performance liquid chromatography (HPLC), thin-layer chromatography (TLC), gas chromatography-mass spectrometry (GC-MS), enzyme-linked immunosorbent assay (ELISA), and biosensor-based screening techniques. The removal of mycotoxins is quite difficult as it is unaffected by physical, chemical, and biological methods [124]. Recently, many studies have shown the potential of Raman spectroscopy and hyperspectral imaging (HSI) techniques for the quality inspection of a variety of food products, representing a promising future for these techniques in the food industry. For the analysis of a broad variety of products, the use of chemometrics with spectroscopy makes these techniques more convenient and effective [125]. The primary goal of food irradiation is to extend the shelf-life of goods stored in diverse circumstances such as stores and houses, as well as to destroy harmful organisms that cause illness as a result of food intake [126]. Electron-beam irradiation (EBI) is a revolutionary food decontamination method that employs low-dose ionizing radiation to eradicate microbial contamination in crops or food. Furthermore, EBI slows crop germination and regulates the ripening rate of vegetables and fruits, increasing the shelf life of these items [127].

### 3.3. Challenges of Analytical Testing

To offer an adequate response to the rising consumer's demands, food analysts have to face increasingly complex challenges that involve using the best available science and technology [128]. Its great potential for rapid identification of trace chemicals has been in surface-enhanced Raman spectroscopy (SERS) history, but the technology is still not ready to be used as a routine analytical method to solve any problems in real-world food analysis, as one of the driving forces for the development of SERS technology is the value of rapid analysis, simplifying sample preparation would continually be one of the major focuses and challenges for applying SERS in food analysis [129].

In NMR spectroscopy, by applying multivariate chemometrics to ^1^H NMR data, instrumental developments allow quick and reliable quantification and authentication of food ingredients simultaneously. However, in ^1^H NMR spectroscopy, signal overlap exists due to its lower spectral resolution and must be carefully considered to measure spectra. The drawbacks of ^1^H NMR spectroscopy include the high initial setup costs, the need for dedicated housing facilities, the provision of cryogens, and dedicated expert personnel [130].

For identifying nanoplastics in food, asymmetric flow field-flow fractionation (AF4) coupled to multiangle light dispersion (MALS) can be used. The technique requires recognizing the nanoplastic particles observed since the LS signal is not selective to a particular type of NPs [131]. The possibility consists of an offline study of the collected fractions of AF4 size by spectroscopy or spectroscopic techniques to classify the eluting nanoplastics. The sensitivity of these techniques, of course, limits this, and because nanoplastics could be present at trace levels, a concentration phase might be needed to ensure proper detection and identification of the material in the collected fractions. For determining the particle absorption and particle concentration in cells, tissues, or small organisms by AF4MALS, fluorescently labeled particles may be used where major problems arise due to autofluorescence from cells/tissues [132].

In identifying food adulteration, mass spectrometry-liquid chromatography (MS-LC) is one of the most commonly used analytical techniques [133]. High-performance liquid chromatography and enhanced ultrahigh performance liquid chromatography have impressive separation capabilities and can isolate and detect many unknown compounds for nontarget detection. The key disadvantages are the relatively low separation in the standard reverse phase setting of hydrophilic compounds and minimal structure knowledge obtained from MS [134].

Biosensors had been hooked up as placing analytical gadgets for fast screening of food impurities, dangerous chemical compounds, and pollutants for food safety. Although biosensors show clear advantages over conventional techniques, a perfect biosensing approach does not yet exist, and there are numerous difficulties in its development to be triumph over. Presently, many biosensors could not without problems have been used for on-website online tracking; consequently, only a few are currently available commercially. Moreover, the weight of the call for the improvement of touchy organic sensing layers has pushed researchers to lay out extraordinarily complicated and luxurious methods, which ultimately grow to be an exceedingly expensive factor [2].

For identifying food adulteration, authenticity, traceability, protection, and consistency, different detection methods utilized include spectroscopic techniques, DNA-based technologies, and immunological techniques, most of which require lengthy and complicated sample preparation and assay times. Ambient mass spectrometry is a new field providing comparable results to other techniques that can overcome these problems. But in terms of quantitation, there are problems regarding how precise the results are and the probability of false-negative and false-positive results. Moreover, the quantification of solid samples cannot be accomplished [135].

Blockchain technology to store chemical analysis data in order is one way to solve traceability problems and ensure transparency as they cannot be manipulated afterward. Although it looks promising, some limits remain to be considered. To scan food tracking data, we mostly rely on sensors, and data collection sensors are linked to the blockchain network. There is no authentication process for the blockchain to prove whether the raw data were correct and has nothing to do if one tempers with a sensor. Also, the overall cost of adopting blockchain technology is uncertain [15].

FT-MIR potential for fingerprinting-based honey authentication is demonstrated which can achieve accuracy levels that could be commercially useful. The combination of multiple vibrational spectroscopic fingerprints in honey authentication is carefully considered in terms of cost/benefit in the industrial context [136]. Electrochemical biosensors have made substantial advances in quantitative detection and screening, and they have enormous potential for overcoming conventional limitations. These biosensors solve the multifactorial food industry's challenge of providing high analytical accuracy amidst complex food matrices while also demonstrating high specificity towards the analyte [137].

Parallel to the consumer's concern about what is in their food and the quality of the food they eat, the development and application of analytical methods and techniques in food science has expanded.

## 4. Food Safety

Foods are being adulterated in various ways. Various hazardous physical, chemical, microbial, and radioactive agents in food cause foodborne diseases. Food safety is a basic element in public health concern, and mycotoxin is a massive food safety challenge worldwide. Food contamination causes a significant impact on food security, trade, economy, and health causing considerable financial losses to the people globally [138]. Food coloring, use of fruit ripening chemicals, mixing of clay, pebbles, sawdust, charcoal decomposed fruits, and vegetables, etc. in food particles are some common methods of food adulteration which may lead to severe diseases like stomach disorder, gastro-intestinal disturbance, liver disorders, kidney failure, cancer, tumor, and toxicity in the body on the consumption of adulterated food [139]. Thus, food safety, food security, and balanced eating are crucial components of food systems that have big repercussions for well-being. Food safety complies with foodborne diseases and encompasses food handling, preparation, and storage [140].

Foodborne diseases worsen individuals' health conditions, and also, they often have detrimental consequences for families, societies, corporations, and ultimately nations. Such illnesses disrupt people's livelihoods by creating a significant impact on healthcare and commerce networks. The Member States of the World Health Organization (WHO) have listed health care, protecting people from immediate potential hazards, as being one of the five core fields of work for WHO in the 12th General Work Programme [141]. The World Health Organization has acknowledged the safety and wholesomeness of irradiated food and advocated its suitable usage as a sanitary treatment. Countries in the Asia Pacific area recognize irradiation as a beneficial method for reducing pathogens of public health relevance as part of overall good manufacturing practice (GMP) and hazard analysis critical control points (HACCP) systems [142]. The food supply chain is influenced by the development, proliferation, or longevity of harmful microbial and chemical agents. That is further associated with the elimination of food waste and the effective usage of natural resources [143].

Food security has been described by the United Nation's Food and Agriculture Organization (FAO) as “a situation that exists when all people at all times have physical, social, and economic access to sufficient, safe, and nutritious food to meet dietary needs and food preferences for an active and healthy life” [144]. The human security concept might also advocate a people-centered approach, assemble on individual capacity, and provide key resources for building resilience in food security and nutrition [145].

**Figure 3:**
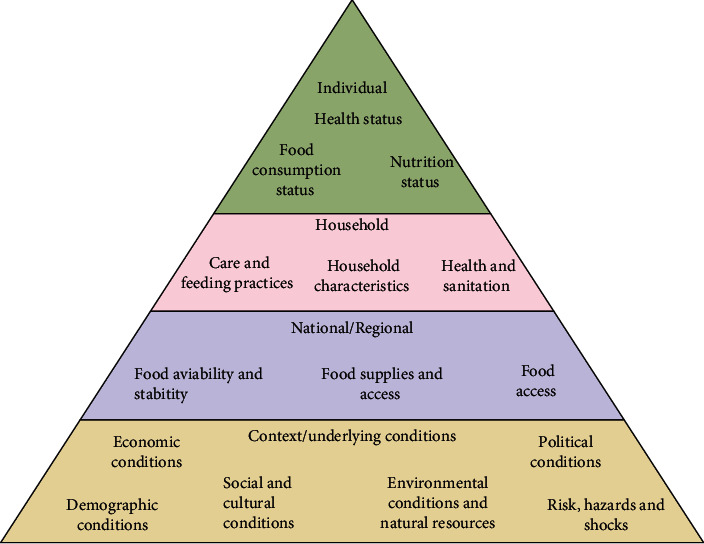
Different levels of food security based on the ideas from the committee on food security and the FIVIMS [147].

According to the Committee on World Food Security (CFS), food and nutrition security are regarded as a time, every individual has physiological, socioeconomic accessibility to food products as well as supplied in adequate quantity and quality to fulfill the nutrition requirements, and that is facilitated by requisite hygiene, healthcare surroundings that ensure a good standard of living [146]. Inter-Agency Working Group (IAWG) has focused on the Food Insecurity and Vulnerability Information and Mapping Systems (FIVMS) that rely on a food diet and nutritional status varying from persons to household to nations which are represented in Figure 3 [147, 148].

### 4.1. Various Aspects of Food Safety

To reduce the risk of foodborne illness, safe food handling methods and protocols are enforced at any point in the food processing life cycle [149]. The different aspects of food safety are summarized as shown in the supplementary section (Figure S2).

#### 4.1.1. Microbiological Aspect

A large number of foodborne diseases and outbreaks have been identified, with pathogenic bacteria, viruses, and protozoa contaminating fresh products and animal products from contaminated sources [150]. Bacterial agents found in food are the main cause of serious and deadly foodborne illnesses [151].

#### 4.1.2. Chemical and Toxicological Aspect

Empirical evidence shows that FCCs can move from food contact materials and articles into food, implying that the vast majority of the human population is exposed to one or more of these chemicals [152]. Heavy metals like lead, arsenic, mercury, cadmium, and copper were found in higher concentrations in some food samples than in others, indicating potential utensil leaching and poor food hygiene [153].

#### 4.1.3. Environmental Aspect

Pesticide residues in the atmosphere are recorded, as well as mass extinctions of nonhuman biota such as bees, birds, amphibians, fish, and small mammals occurred due to food spoilage [154]. The Stockholm Convention, which was accepted in 2002, outlawed persistent and bioaccumulative chemical compounds like DDT, toxaphene, HCH, aldrin, and dieldrin and substituted them with environmentally safe and less-bioaccumulative chemicals [40].

#### 4.1.4. Nutritional Aspect

Diets are having devastating health effects when combined with snacking behavior associated with busy lives and increasingly sedentary behaviors, and the burden of illness due to diets and lifestyles may well raise more throughout the future. For goods that may not come under the EU's existing diet and health claim regulations, or similar legislative structures around the world, a regulatory mechanism may be required [155, 156].

#### 4.1.5. Personal Hygiene

Food handlers and preparers with poor personal grooming habits put their own and the public's health at risk. Many foodborne illnesses can be avoided with simple practices including extensive hand washing and proper washing facilities [157].

#### 4.1.6. Legislative Aspect

Food legislation's main goals are to protect the consumer's well-being, protect the consumer from theft, and promote trade. It may also be appropriate to pass legislation prohibiting the adding of nutrients to foods where it is nutritionally unnecessary or unhealthy or where fortification may give the wrong opinion about nutrition [158]. The EFSA, European Food Safety Authority, in the European Union (EU), and the FDA, Food and Drug Administration of the United States of America, are the three most powerful regulatory bodies in the world that legislate and enact the legislation and supervise clearance and control of food additives. The JECFA, the Joint Food, and Agriculture Organization (FAO)/World Health Organization (WHO) Expert Committee on Food Additives, and the Codex Alimentarius are two other relevant organizations that assess safety risks, conduct research, question statements, and are generally concerned with food additives [159]. Hazard Analysis Critical Control Point system (HACCP) and other quality assurance systems like ISO 9000 system can be used to assure the quality of both goods (products) and services. The ISO 9000 family includes standards such as the ISO 9001:2015, the ISO 9000:2015, the ISO 9004:2009, and the ISO 19011:2011 to monitor food hygiene [160].

Food supply, sustainability, utilization, and safety are prominent food security features within the food supply chain. A horrible situation of illness and food insecurity hampering every age group created by food contamination. A collaborative approach among government agencies, manufacturers, distributors, and purchasers will undoubtedly help ensure food safety.

## 5. Biodegradable Food Packaging Materials

Materials for the packaging of foods commonly include plastics, sheets, glasses, and metals such as aluminum foils, laminates, and tin-plates. Packaging materials protect food from degradation by providing various mechanisms, such as avoiding entry to the product, odor transmission prevention, and the conservation of an internal packaging environment [161]. Plastics such as polyvinyl chloride (PVC), polyethylene (PE), and polyamide (PA) have high thermal tolerance. They are inexpensive and have exceptional mechanical characteristics, such as carbon dioxide and oxygen, and heat tolerance. Plastics are, therefore, used intensely for food packaging [162]. As a result of food packaging, a massive amount of plastic waste is formed, and its management turns into a global problem in many developing and underdeveloped countries [17]. However, the biodegradable packaging material is scattered as small fragments into the biodegradation waste and byproducts and formed carbon dioxide, water, and biomass, which is recycled to the natural environment by biocycles by microorganisms [163]. Polyhydroxyalkanoates (PHAs) that can be prepared from renewable and biowaste resources are considered as biodegradable and biocompatible biomaterials and prospective replacement for nondegradable plastics [164].

**Figure 4:**
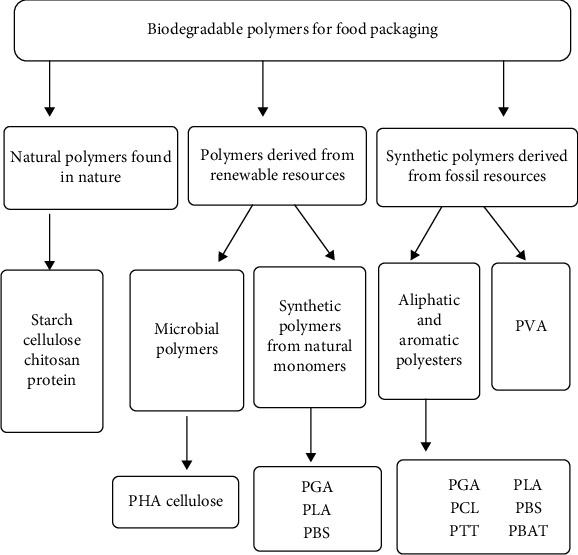
Classification of biodegradable polymers for food packaging application [165].

Many natural and synthetic polymers that have been found biodegradable are used for food packaging applications. Natural polymers like cellulose, starch, and protein are used in food packaging, etc. Likewise, fossil fuel sources such as gasoline, natural gas, and coal, as well as natural monomers, are used in food packing, and all of which are naturally biodegradable, originate synthetic polymers as shown in Figure 4 [165].

### 5.1. Based on Natural Polymers

Synthetic polymers are the most widely used materials for packaging because of their ease of processing, low cost, and low density. However, many of these materials are not easily recyclable and are difficult to degrade completely in nature, creating environmental problems. Thus, there is a tendency to substitute such polymers with natural polymers and copolymers that are easily biodegraded and less likely to cause environmental pollution. There has been a greater interest in poly-lactic acid (PLA), polyhydroxyalkanoates (PHAs), cellulose and starch-based polymers, and copolymers as the emerging biodegradable material candidates for the future. The field of use of biodegradable polymer in food-contact articles incorporates expendable cutlery, drinking cups, serving of mixed greens cups, plates, overwrap and cover film, straws, stirrers, covers and cups, plates, and holders. Cellulose is the most bountiful, practical, compostable, biodegradable, and reusable natural material on earth and has numerous applications. Plant cellulose is dominatingly accepted to be among the most extravagant natural polymers on earth. Paper and board materials have great mechanical properties as bundling material; however, the gas and water fume porousness is regularly exceptionally high for some food applications [166].

Poly (lactic acid) (PLA) is one of the most encouraging biopolymers acquired from the controlled depolymerization of the lactic acid monomer obtained from the fermentation of sugar feedstock, corn, etc., which are renewable resources readily biodegradable. It is a flexible polymer, recyclable, and compostable, with high transparency, high molecular weight, great processability, and water solvency opposition [167].

In addition to minimizing environmental contamination as biodegradable, edible protein packaging films often improve packaged food quality, including tastes and colorings. In the development of biodegradable films that can preserve food by controlling the bacterial formation, naturally occurred compounds, including nisin, pediocin, or lysosomes, are used [168]. Composite biofilms are made of two types of biomolecules, namely, hydrocolloids and lipid. Since composite films are an obstruction to oxygen, water, and carbon dioxide, food products' fragrance is preserved. They are therefore used in food packaging [169].

The most plentiful polysaccharide on earth after cellulose is chitosan. Chitosan is created from chitin by deacetylation to eliminate the acetyl group [170]. The chitosan-based composite nanolayers can be used to develop novel food packaging materials that can potentially be as functional as conventional plastics and, therefore, replace conventional plastic packaging while leaving a significantly lower environmental footprint [171]. The incorporation of different nanomaterials into biobased polymers such as chitosan, potato starch, carboxymethyl cellulose (CMC), cornstarch, and Arabic gum can improve the different properties of packaging materials by enhancing antimicrobial activity, thus preventing foodborne pathogens, thereby significantly enhancing the properties of biobased materials like food packaging materials [172]. Numerous examples show that bacterial growth can be inhibited by organic acid-based food packaging and extend foods' shelf life. Researchers developed antimicrobial EVOH films with sorbic acid chitosan microcapsules (S-MPs) and applied them to fish fillets (Hu et al., 2017). In terms of the antimicrobial property, TiO_2_ nanoparticles (NPs) were found to kill a wide range of microorganisms, including Gram-negative and Gram-positive bacteria, fungi, protozoans, and virus bacteriophage [173].

Organic acids are commonly used as traditional food additives, including propionic acid, lactic acid, malic acid, sorbic acid, and tartaric acid. Sorbic acid and potassium sorbate are active against many bacteria and molds [129]. Due to their potential in the food and pharmaceutical industries and aromatherapy, essential oil (EOs) kits have shown in vitro efficacy against microorganisms and oxidants in food experiments and/or food simulator evaluation tests [174]. The possible use of natural and sustainable ingredients, rather than conventional synthetic molecules or chemical compounds, is being used in packaging systems. Nanotechnological methods are promising techniques for the entire agricultural industry chain, from industry to customers [175].

Adding antimicrobial properties to the biodegradable packages, these new materials can offer enhanced protection against food spoilage, extending the shelf life [176]. Because of the low priced, nontoxic, antibacterial action, poly (butylene adipate-co-terephthalate) films incorporating different amounts of chitosan nanofibers have great potential in food and pharmaceutical packaging [177]. Nanocomposite films based on cellulose acetate and polyethylene glycol cetyltrimethylammonium bromide-modified montmorillonite can be used as an active packaging material because of its good antimicrobial activity as well as nontoxicity on human blood [178].

### 5.2. Based on Polymers Derived from Renewable and Fossil Resources

Petroleum-based plastics are the most commonly used polymers in packaging applications due to their superior properties and relatively low cost [179], although these plastic materials are not biodegradable, recycled, and depend on nonrenewable sources leading to adverse environmental depletion and global warming [180]. To overcome these problems, biodegradable polymers derived from renewable resources are given much attention in recent years for the packaging process, which is driven by increased public awareness of the global environmental challenges related to nondegradable plastics. Biodegradable polymers for food packaging from a renewable source can be classified as microbial polymers and synthetic polymers from natural monomers [181].

### 5.3. Microbial Polymers

Microbial polymers are obtained from microorganisms through the metabolic engineering process. Polylactic acid (PLA), polyhydroxyalkanoates (PHA), and exopolysaccharides (EPS) are the major microbial fermentation-based biopolymers [182].

Polyhydroxyalkanoates (PHA) are obtained from renewable raw materials like fatty acids, maltose, and glucose from biotechnological conversion [183]. PHAs are biocompatible, crystalline, and nontoxic thermoplastic elastomers with a low melting point, good UV resistance, and physical and chemical properties that depend on the composition of PHA monomer [184]. Polyhydroxybutyrate (PHB) is the common illustration of PHA for short-term food packaging applications with a high degree of crystallinity. It has the benefits of biodegradability with 70% crystallinity showing mechanical properties like polyethylene. Additionally, PHB is used in packaging applications because of its lamellar structure and higher aroma barrier properties with the permeability of water vapor [185].

Exopolysaccharides (EPS) are complex microbial polymers synthesized from bacteria, fungi, and blue-green algae. They are composed of carbohydrates and are secreted outside the cell wall [182]. Among various types of EPS like alginate, glucans, dextrin, and xanthan, kefiran gains much attention in packaging application due to its water solubility, biocompatibility, emulsifying and stabilizing effect, and biodegradability [186].

### 5.4. Based on Synthetic Polymers from Natural Monomers

Synthetic polymers from natural monomers for food packaging technology are novel biodegradable and eco-friendly, alternative to petrochemical plastics and synthetic polymers from fossil resources.

Polyglycolic acid (PGA) is a biodegradable polymer obtained from both petrochemical resources and renewable-derived monomers. It is a rigid polyester used in packaging applications as a protective layer for carbonated soft drinks and beer [187]. PGA possesses a similar structure as PLA but retains higher heat distortion temperature, high crystallinity, mechanical properties, biocompatibility, and gas barrier properties against carbon dioxide and oxygen [188]. On the other hand, large-scale production and application of PGA are still facing challenges due to the lack of a low-cost method for monomer preparation [189].

Polylactic acid (PLA) is a biodegradable polymer obtained from lactic acid by the fermentation of renewable crops like starch, sugar, and corns. It has gained much attention in food packaging because of its comparatively better mechanical strength, easy availability, durability, and transparency than other biodegradable plastics [188, 190]. Due to the high gas permeability and poor barrier property, PLA is not applicable for beverage bottle applications, which can be improved by combining with high barrier plastics like PGA from melt compounding or lamination [188].

Poly butylene succinate (PBS) is an aliphatic polyester prepared from the polycondensation of succinic acid and 1,4-butanediol monomers with noble biodegradability, compostability, extensive thermoplastic processing, stable mechanical properties, and good thermal and chemical resistance. Nowadays, these monomers can be produced from renewable biomass like starch, xylose, and glucose [191]. PBS is used in the food packaging application because of its resistance towards degradation when exposed to heat and light [192]. The properties of PBS like high crystallinity, good thermal properties, mechanical properties, and easy processibility made these polyester candidate materials for food packaging applications such as films and semirigid bowls [193, 194]. The low impact strength and tear resistance might limit its application in food packaging [195].

Even though these polymers derived from renewable sources are biodegradable, biostable, environment friendly, and mostly used as packaging material, they limit in their large-scale industrial applications due to oxygen, water vapor barriers, thermal heat resistance, and expensive cost [196].

Biodegradable polymer advancement is yet in its initial stages; as of now, it covers a little part of the current bundling market around the world. Biopolymers satisfy the ecological concerns; however, such have a few constraints regarding financial aspects and execution.

## 6. Challenges and Suggestions

One of the most challenges for the present and future is the supply of sufficient, healthy, pure, and safe food to the increasing world population. Among the world population, almost 11% of them are still undernourished despite sufficient energy supply [197]. Food security, the access to sufficient amounts of food, cannot be achieved by growing agricultural production or fair global distribution alone since most food is wasted between production and retail. Hence, developing innovative technologies for food preservation, processing, and packaging is required. But the sophisticated analytical techniques used for food control generally work in a targeted mode where contamination remains undiscovered, raising the level of contaminants [198].

The presence of various toxigenic fungi or their secondary metabolites as mycotoxins in human food and livestock feed is a recurring food safety problem so the mycotoxin-producing species and the particular strain must be authentically identified by adopting recently developed techniques (like PCR-based techniques, laser biospeckle technique, apta-sensor, immune-sensor, enzymatic-sensors, and others) and by the development of new fungal selective agar media for the isolation of toxigenic fungal strains only. Similarly, several innovative methods such as attenuated total reflectance Fourier transform infrared spectroscopy (ATR-FTIR), enzyme-linked immunosorbent assay (ELISA), liquid chromatography-tandem mass spectrometry (LC-MS/MS) via a multiple antibody immunoaffinity column, and suspension array technology can be applied for detection of mycotoxins in commodity [199, 200]. As shown by recent reports, “Surface Active Maghemite Nanoparticles” is indicated as a stable and good magnetic nanocarrier for mycotoxin removal; the potential of which can be tapped by the food industries [201]. Food chemistry has an important task to improve food security to give perishable products longer shelf lives by developing appropriate strategies.

## 7. Conclusions

Food can be contaminated, often by different variants such as microorganisms, heat, moistness, or water, contributing to many foodborne diseases. Food safety and security are important to maintain the wellness of human beings. Thus, to ensure food safety, biodegradable packaging materials are exclusively used since they are eco-friendly. The procurement of suitable packaging materials and technology plays a crucial role during the delivery and storage process to properly preserve quality and freshness. Food adulteration entails incorporating irrelevant, deleterious chemicals into food, which diminishes the food's quality and has become a significant problem to people's well-being. Though a massive number of analytical tools and techniques are used for food analysis, there are still a decent number of problems that need to be improved. People around the globe have changed their diets from mainstream food for the increment of the immune system. To meet the ever-increasing food demand, alternative food sources of high nutritional value, such as snails and home processed food, are considered a good alternative candidate. The rationale of this article was to present the main chemical reactions that are present in food, for instance, Maillard reaction, caramelization reaction, oxidation reaction, and formation of acrylamide in the various food matrices and demonstrate the need for modern analytical techniques and numerical analyses to elucidate the various reaction pathways that are in some cases still very complex to understand in depth. Understanding these reactions enables us to put forward mitigation strategies in food processing technology to limit the occurrence of toxic or carcinogenic compounds.

## Data Availability

The data used and/or analyzed in the study are available from the corresponding author on reasonable request.

## Supplementary Materials

Supplementary Materials Figure S1: Maillard reaction scheme adapted from Hodge. Figure S2: aspects of food safety.
