# Supplementary material for: Current Trends of Food Analysis, Safety, and Packaging
Source: Int J Food Sci. 2021 Aug 24;2021:9924667. doi: 10.1155/2021/9924667 (PMC8410450; doi:10.1155/2021/9924667)
Supplement: Supplementary Materials — Figure S1: Maillard reaction scheme adapted from Hodge. Figure S2: aspects of food safety. [file 9924667.f1.docx]

## **Current trends of Food analysis, Safety, and Packaging**

Bindu Modi^1^, Hari Timilsina^1^, Sobika Bhandari^1^, Ashma Achhami^1^, [Sangita Pakka](https://www.facebook.com/sangita.pakka?comment_id=Y29tbWVudDoyNzU5NjUwMzQwOTQ2MjMwXzI3NTk2OTA3Njc2MDg4NTQ%3D)^1^, Prakash Shrestha^1^, Devilal Kandel^1^, Dhan Bahadur GC^1^, Sabina Khatri^1^, Pradhumna Mahat Chhetri^2^ and Niranjan Parajuli^1*^

*^1^Biological Chemistry Lab, Central Department of Chemistry, Tribhuvan University, Kirtipur, Kathmandu, 44618, Nepal*

*^2^Department of Chemistry, Amrit Campus, Tribhuvan University, Leknath Marg, Kathmandu, 44600, Nepal*


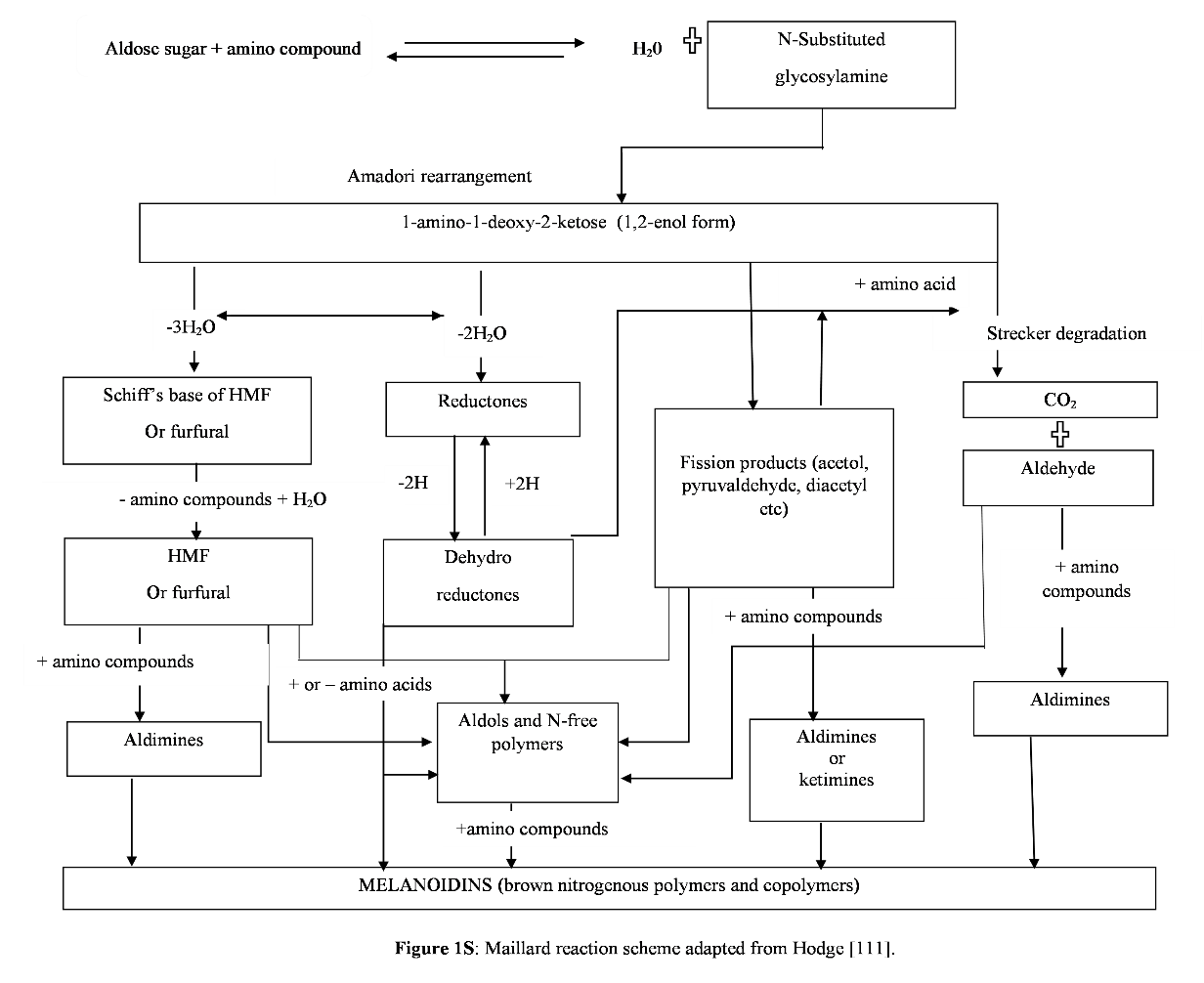


**Figure S1** Maillard reaction scheme adapted from Hodge.


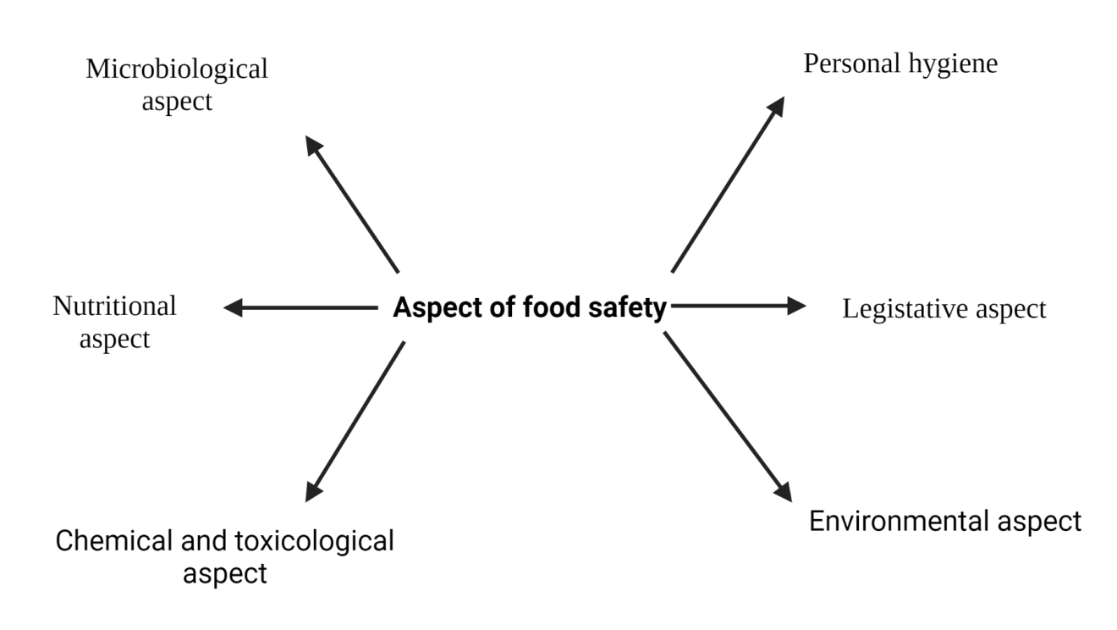


**Figure S2** Aspects of food safety
